# Discovering miRNAs Associated With Multiple Sclerosis Based on Network Representation Learning and Deep Learning Methods

**DOI:** 10.3389/fgene.2022.899340

**Published:** 2022-05-17

**Authors:** Xiaoping Sun, Xingshuai Ren, Jie Zhang, Yunzhi Nie, Shan Hu, Xiao Yang, Shoufeng Jiang

**Affiliations:** ^1^ Department of Neurology, Zhenhai People’s Hospital, Ningbo, China; ^2^ Department of Respiratory, Zouping People’s Hospital, Binzhou, China; ^3^ Department of Neurology, Zouping People’s Hospital, Binzhou, China; ^4^ Nursing Department, Second Sanatorium of Air Force Healthcare Center for Special Services, Hangzhou, China; ^5^ The Center of Physical Therapy and Rehabilitation, Zhejiang Hospital, Hangzhou, China; ^6^ Department of Neurology, Shulan Hangzhou Hospital, Hangzhou, China

**Keywords:** multiple sclerosis, deep learning, disease related miRNAs, miRNA discovery, network representation

## Abstract

Identifying biomarkers of Multiple Sclerosis is important for the diagnosis and treatment of Multiple Sclerosis. The existing study has shown that miRNA is one of the most important biomarkers for diseases. However, few existing methods are designed for predicting Multiple Sclerosis-related miRNAs. To fill this gap, we proposed a novel computation framework for predicting Multiple Sclerosis-associated miRNAs. The proposed framework uses a network representation model to learn the feature representation of miRNA and uses a deep learning-based model to predict the miRNAs associated with Multiple Sclerosis. The evaluation result shows that the proposed model can predict the miRNAs associated with Multiple Sclerosis precisely. In addition, the proposed model can outperform several existing methods in a large margin.

## Introduction

Multiple sclerosis is a central nervous system disease that affects a lot of young adults worldwide. The amount of patients with Multiple Sclerosis is increasing in both developed and developing countries(Amoruso, 2020 #5) (Browne et al., 2014) The biological basis and underlying cause of Multiple Sclerosis are still unknown. Some existing studies show that Multiple Sclerosis is associated with several genes or other genetic biomarkers, which may increase the disease susceptibility (Ebers, 2008). It is a challenge to discover pathogenesis and disease-related biomarkers. Discovering such biomarkers will effectively contribute to studying the biological mechanisms of Multiple Sclerosis and will help people to understand Multiple Sclerosis. To improve efficiency, computational methods have been designed for identifying biomarkers of Multiple Sclerosis (Bielekova and Martin, 2004; Ziemssen et al., 2019). However, most existing methods for discovering biomarkers of Multiple Sclerosis focus on identifying the disease genes. Few of them are focused on identifying the miRNA related to Multiple Sclerosis. Since it has been shown that miRNAs could be biomarkers of Multiple Sclerosis (Amoruso et al., 2020), it is important to develop a novel algorithm to identify the disease-related miRNAs of Multiple Sclerosis rapidly and effectively.

Although not too many methods are designed for identifying biomarkers of Multiple Sclerosis, identifying biomarkers of diseases has attracted a huge amount of attention in recent years. Several computational methods have been developed for predicting disease genes since such methods are helpful for saving time and money. The Guilt-by-association hypothesis is the basis of most of the proposed methods for predicting disease-related genes. In detail, the hypothesis is that genes related to the same disease may have a higher probability of having the same topological structure in the protein-protein interaction network. For example, genes associated with the same disease may be neighbors or in the same clique in the protein-protein network. Therefore, based on the guilt-by-association hypothesis, one of the key problems of predicting disease-related genes is how to measure the similarity between known disease-related genes and candidate genes precisely. By now, a lot of approaches have been developed to compute similarities between genes. One of the earliest approaches is to directly count the neighborhoods (Oti et al., 2006), which counts the number of disease-related genes in their neighborhoods in the protein-protein network. Given a gene g, if most neighbors of g are associated with the disease, gene g has a high probability to associate with the disease. This approach ignores these disease-related genes which are not direct neighbors of g in the protein-protein-interaction network.

To overcome this drawback, some proposed approaches use the shortest path-based model to measure the distance between genes (Krauthammer et al., 2004). However, these methods did not perform well in some cases. The reason is that both aforementioned methods only take the local topological structure of the protein-protein interaction network into account, ignoring the global topological information of the protein-protein network. A lot of studies have shown that considering global topological information would be able to improve the performance of disease gene prediction (Ma et al., 2016). Therefore, to consider the global topological information, several studies have tried to use random walk with restart to capture the global topological information (Valdeolivas et al., 2019). Furthermore, other network representation methods, like node2vec, are used to predict disease (Li et al., 2021). However, these methods are not designed for predicting the miRNA associated with Multiple Sclerosis. In addition, it is a challenge to consider both the gene feature and miRNA feature for Multiple Sclerosis-associated miRNA prediction. Inspired by existing research and to overcome the challenge, we aim to propose a method to predict Multiple Sclerosis-associated miRNAs based on network representation and deep neural networks.

In this study, we propose a novel computation framework for predicting Multiple Sclerosis-associated miRNAs. The framework firstly learns the feature representation of miRNA based on a network representation model. Then, a deep learning-based framework is used to predict the miRNA associated with Multiple Sclerosis. The main contributions of this study can be listed as follows:

1) A network representation learning-based method is proposed for learning the feature representation of miRNA based on a protein-protein interaction network and a miRNA-gene regulation network.
2) A convolution neural network-based model is proposed for predicting Multiple Sclerosis-associated miRNAs based on the low-dimensional features learned based on the network representation learning-based method.
3) The evaluation shows that the proposed model can predict Multiple Sclerosis-associated miRNAs precisely.

## Materials and Methods

### Construction of miRNA Networks

In order to predict MS-related miRNAs based on the hypothesis of guilt-by-association, we first construct two miRNA-related networks: miRNA-mRNA interaction network and miRNA functional similarity network. We obtain the miRNA targeted mRNA interactions of human from the mirTarBase database (Hsu et al., 2011), which records experimentally validated miRNA-target interactions. There are mainly six types of experimental evidence supporting the miRNA-target interactions, including western blot, luciferase assay, pSILAC, microarray, NGS, and CLIPseq. In this work, 380,639 miRNA-mRNA interactions are downloaded and used to construct the heterogenous bipartite RNA network, covering 2,599 miRNAs and 15,064 mRNAs.

The previous study has demonstrated that miRNAs with similar functions are more likely associated with the same disease (Wang et al., 2010; You et al., 2017). In this work, we use a miRNA functional similarity network calculated by MISIM (You et al., 2017). In detail, MISIM measures the functional similarity between two miRNAs by measuring the semantic similarity of their associated diseases while considering the structures of disease relationships. We use a well-constructed miRNA functional similarity score matrix from You et al. (You et al., 2017), which consists of 495 miRNAs. In this miRNA functional similarity symmetric matrix, each matrix element indicates how are the corresponding miRNAs functionally similar to each other. In the following step, we will learn miRNA features based on the two miRNA networks.

### Extract miRNA Features From Networks Using Graph Embedding Technique

Before predicting MS-related miRNAs, we first extract features of miRNAs from the miRNA functional similarity network and miRNA-mRNA interaction network. Based on the hypothesis that miRNAs with higher similarity to known disease-related nodes are more likely to be disease-associated, we mainly extract node features based on the global network topological structure. In this work, we use a widely applied graph embedding technique, named Node2vec (Grover and Leskovec, 2016), to extract the topological features of nodes in a network. Node2vec (Grover and Leskovec, 2016) is a graph embedding or representation method by extending DeepWalk (Perozzi et al., 2014). It features in finding neighborhood of a node using both deep-first-search (DFS) and breath-first-search (BFS) in the random walk strategy. Specifically, Node2vec first generate multiple random-walk paths with fixed length for each node in a network. Node2vec applies a biased random walk strategy using return parameter (p) to control the probability of walking steps to previous visits, and in-out-parameter (q) to control the probability of walking steps to directions more deeply (DFS) or widely (BFS). Particularly, we set the in-out-parameter (q = 0.5) return parameter (p = 10) and other random walk-related parameters in default.

The node feature vectors learned under this strategy have two important characteristics: First, Homophily, that is, nodes in the same community have similar feature vectors; Second, structural equivalency, that is, nodes with similar structural characteristics (even without directly connected edges) have similar feature vectors. Next, our method uses skip-gram neuron network model (Guthrie et al., 2006; Church, 2017) for data training, during which stochastic gradient ascent method and negative sampling strategy are used to efficiently fit the data. In detail, given node u in a network G(V, E) , suppose ${\text{N}}_{\text{S}}\left(\text{u}\right)$ represents neighborhoods of node u under the random-walk strategy S , the purpose is to find an encoder function $\mathrm{f:f}\left(\text{u}\right)\in {\mathbb{R}}^{\text{d}}$ , where d is the size of feature dimension. The optimization purpose is to maximize the objective function shown in Eq. 1. Equivalently, the loss function can be represented as Eq. 2, where P(v|f(u)) can be calculated using Eq. 3 (in form of softmax function), which can be further simplified using negative sampling strategy (Grover and Leskovec, 2016).

$$\text{m}\text{a}{\text{x}}_{\text{f}}\sum_{\text{u}\in \text{V}}\log P\left({\text{N}}_{\text{S}}\left(\text{u}\right)|\text{f}\left(\text{u}\right)\right)$$ (1)

$$ℒ=\;\sum_{\text{u}\in \text{V}}\sum_{\text{v}\in {\text{N}}_{\text{S}}\left(\text{u}\right)}\mathrm{-log}\left(\text{P}\left(\text{v}|\text{f}\left(\text{u}\right)\right)\right)$$ (2)

$$\text{P}\left(\text{v}|\text{f}\left(\text{u}\right)\right)=\frac{\exp\left(\text{f}{\left(\text{u}\right)}^{\text{T}}\text{f}\left(\text{v}\right)\right)}{\sum_{\text{n}\in \text{V}}\exp\left(\text{f}{\left(\text{u}\right)}^{\text{T}}\text{f}\left(\text{n}\right)\right)}$$ (3)

For miRNA functional similarity network, the edges are weighted by miRNA similarities, and the edge weights affect the random walk process with the transition probabilities proportionate to the weights. For miRNA-mRNA interaction network, we simplify the network as a homogeneous unweighted network without considering the node types. Only miRNA features are used in the downstream prediction task. As a result, we generate miRNA features with 512 dimensions in both networks.

### Convolutional Neuron Network-based Prediction Framework

**FIGURE 1:**
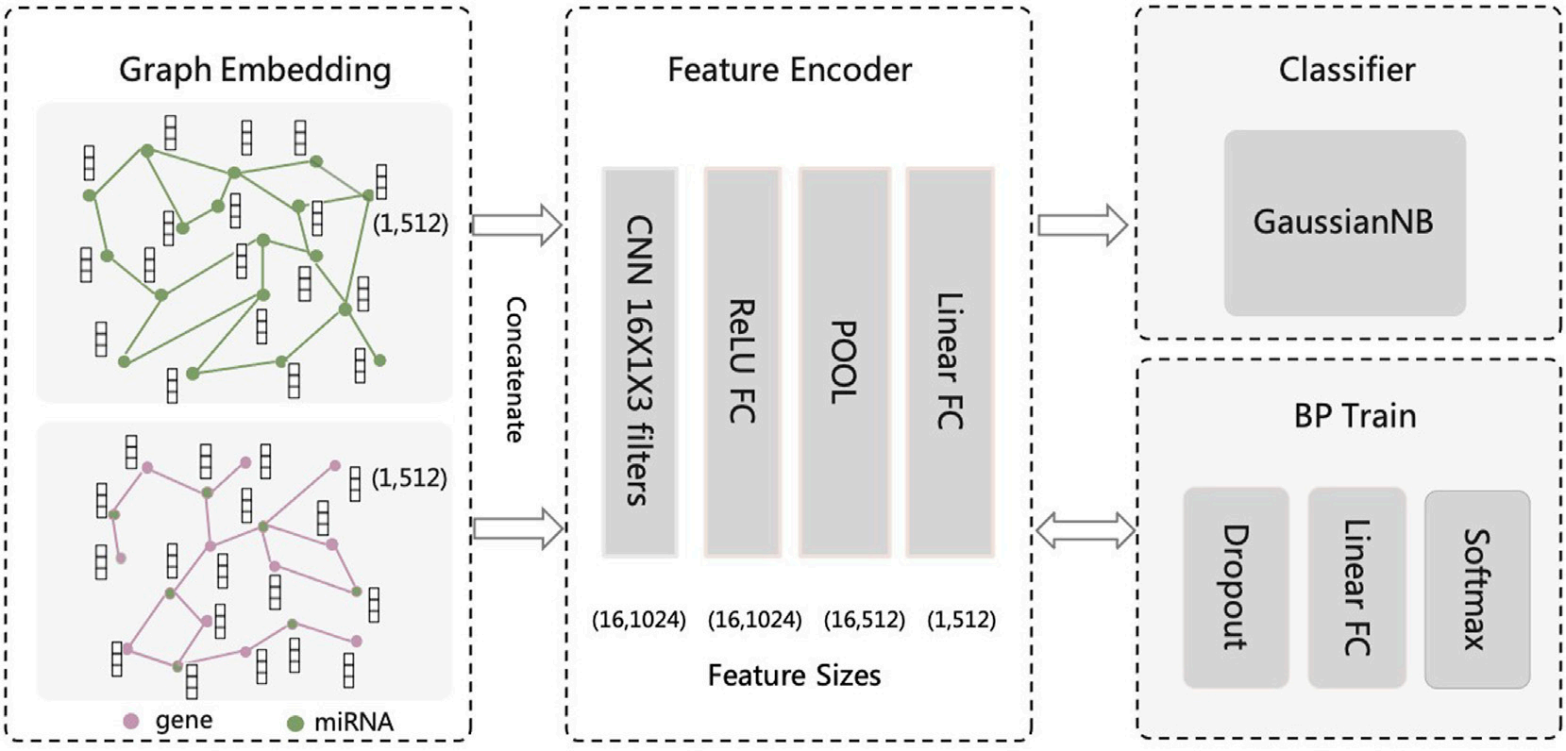
The CNN-based computational framework for predicting miRNA associated with Multiple Sclerosis.

We construct a convolutional neuron network-based model to further fuse miRNA features and predict MS-related miRNAs. As shown in Figure 1, our framework can be divided into three parts: feature encoder, backpropagation (BP) training with dropout, and Gaussian Naive Bayes (GaussianNB) classifier. The BP training part is used to train the CNN-based feature encoder model. The novelty of our workflow is that we use CNN to encode the miRNA features, while using traditional classifier (i.e., GaussianNB) for the prediction task.

The feature encoder first transforms features using a convolution layer with 16 one-dimensional convolution kernels of size 1 × 3. The input feature of each miRNA is concatenated from separate features from two networks mentioned above (i.e., feature size equals 1024). If miRNA only exists in one network, the miRNA feature will be padded by zero values. The CNN layer is followed by a full connection (FC) hidden layer with Relu as activation function. The FC hidden layer has the same neuron size as the CNN layer output (i.e., 16 × 1024). Next, a max-pooling layer with filter size of 1 × 2 and step size of 2, and a linear FC layer with 256 neurons is followed, leading to a feature map of size 1 × 256. Next, a dropout layer with 50% dropout probability, a linear FC layer with 2 neurons, and a softmax layer is followed by the feature encoder part for model BP training.

The function of the max-pooling layer is to perform a downsampling process on the feature map. It has no parameter weights and is simple to calculate, but it can reduce dimensionality features, reduce the number of parameters, increase nonlinearity, prevent overfitting and improve the generalization ability of the model. For BP training of convolutional neural network, we introduce fully connected layer and softmax function as the classifier during training and use dropout to make the training more efficient. The dropout layer reduces overfitting by failing half of the hidden layer neurons to stop working during forward propagation. This method can reduce the interaction between hidden layer nodes and reduce the interdependence between hidden layers. Reducing the redundancy of the intermediate layer of features will increase the orthogonality between the various features of each layer, which can lead the model more generalizable.

Instead of using the softmax layer as the final classifier, we extract the features output from the first linear FC layer, and use a traditional classifier, namely Gaussian Naive Bayes (GaussianNB), for the prediction task, because only a small number of positive cases (MS-related miRNAs) exist in our sample. All parameters in GaussianNB are used in their default values. In the experiment results, we will show our design of the framework is capable to predict MS-related miRNA accurately.

### Metrics for Performance Evaluation

To evaluate the performance of our proposed model, and compare it with other methods, we use five-fold cross-validation and three widely-used matrices (i.e., ROC-AUC, PR-AUC, and F1-score) for performance evaluation. ROC-AUC estimates the area underlying the receiver operator characteristic curve, and it summarizes the trade-off between the true positive rate and the false positive rate. PR-AUC estimates the area underlying the precision and recall rate curves, and it summarizes the trade-off between true positive rate and positive predictive rate. The precision represents the proportion of all predicted true positive samples that are predicted to be positive, and the recall rate measures the proportion of actual positives that are identified correctly. F1-score is the harmonic mean of precision and recall, which can simultaneously reflect the precision and recall of a prediction model.

PR-AUC and ROC-AUC perform differently when dealing with unbalanced samples. The PR-AUC curve is sensitive when the data is unbalanced and changes strongly as the proportion of positive and negative samples changes. However, the ROC-AUC curve is less sensitive towards the ratio of positive and negative sample sizes. ROC-AUC is always applied to the balance of observations between each class, while PR-AUC is better matric when evaluating cases of imbalanced datasets.

## Results and Discussion

### Dataset for Experiment

We download the miRNA-multiple sclerosis associations from the disease-related miRNA database named HMDD (Li et al., 2014), which is a manually collected database with experiment-supported evidence (http://www.cuilab.cn/hmdd). 102 MS-related miRNAs are extracted from the database as positive samples. Previous works usually randomly select the negative samples from the unlabeled disease associations (Peng et al., 2019; Wang et al., 2021). And they usually select a collection of negative samples with size equal to the positive samples. However, there are usually many more negative samples than positive samples in disease gene prediction because only a few genes are associated with the disease.

In this case, we randomly selected associations with n times the number of positive samples from the unlabeled miRNA-MS associations as negative samples in our experiments, where n∈(2,10,20,30,40,50). And we also tested the performance using all unlabeled miRNA-MS associations as the negative samples. We use five-fold cross-validation and AUC-ROC, AUC-PR, and F1-score as evaluation matrices (Kohavi, 1995).

### Performance of Proposed Framework in Predicting MS-Related Genes

We first evaluate the performance of our proposed framework in the task of predicting MS-related genes. In this experiment, we use n times the number of positive samples from the unlabeled miRNA-MS associations as negative samples in our experiments, where n∈(2,10,20,30,40,50). And we also tested the performance using all unlabeled miRNA-MS associations as the negative samples.

**FIGURE 2:**
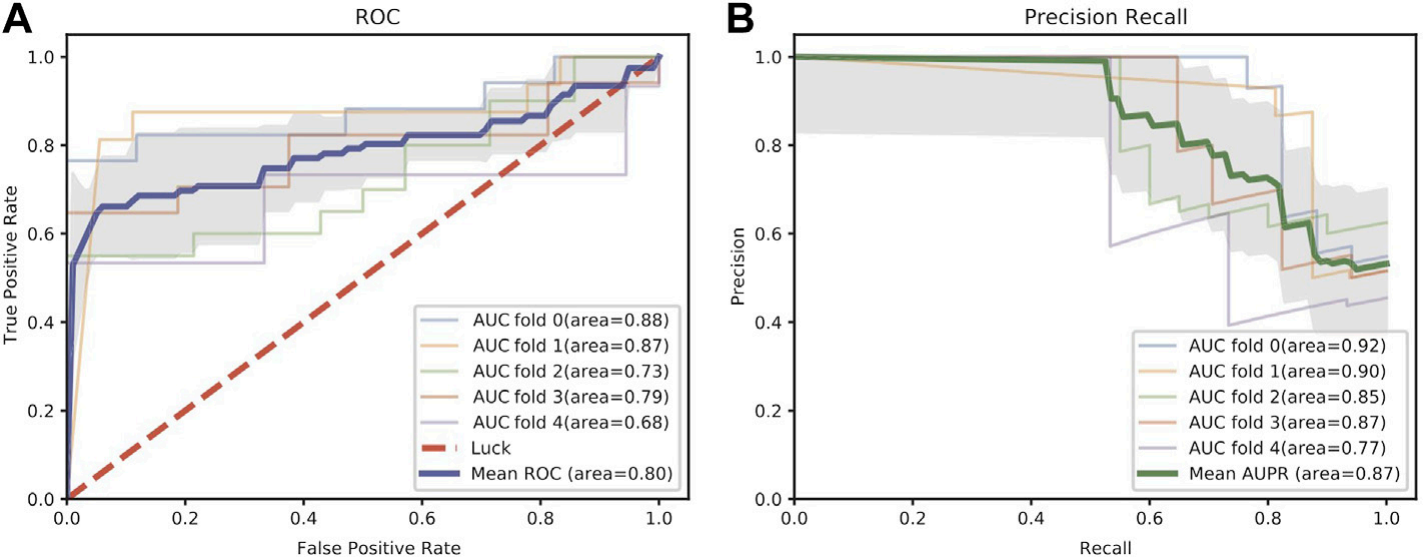
ROC **(A)** and PR **(B)** curves in five-fold cross-validation of the miRNA-disease association prediction task. The shaded area means the estimated standard deviation of ROC and PR curves around the mean across five-fold cross-validation.

Figure 2 shows the receiver operator characteristic (ROC) curve and precision-recall (PR) curve using two times the number of positive samples from the unlabeled miRNA-MS associations as the negative samples. The mean ROC-AUC reaches 0.8 and the mean AUPR reaches 0.87 across five-fold cross-validation. We also evaluate the prediction performance as the number of negative samples increases. As shown in Table 1, the mean ROC-AUC remains relatively stable and even increases to 0.87 when using all negative samples, which demonstrates the predictive ability of our proposed model. As expected, as the size of negative samples increases, the mean PR-AUC and mean F1-score gradually decrease. Even though, we think using the “all negative samples” is more similar to the real case when measuring the performance of prediction models. In the following experiments, we will use “all negative samples” to evaluate the performance.

**TABLE 1:** Average ROC-AUC, PR-AUC, and F1-score as the size of negative samples increases.

Negative sample size*	Mean ROC-AUC	Mean PR-AUC	Mean F1-score
2×P	0.8060	0.8704	0.7849
10×P	0.7287	0.6469	0.6110
20×P	0.7745	0.6193	0.5739
30×P	0.8631	0.6438	0.5615
40×P	0.8774	0.6217	0.5531
50×P	0.8623	0.5594	0.5285
All	0.8696	0.4110	0.2693

*P represents the size of positive samples, in our case P = 102; and All represents all unlabeled miRNA-MS associations as the negative samples.

### Comparison With State-of-the-art Methods

We evaluate the performance of our proposed methods and four widely used machine learning methods (decision tree, SVM, logistic regression, and GaussianNB) on the task of miRNA-MS association prediction, all unlabeled MS-related miRNAs as the negative sample, i.e., unbalanced testing data. The four methods we compared are all implemented in the python package scikit-learn, and their default parameters are used in this experiment. For fair comparisons, all of the four methods use the same miRNA features as our model extracted from the two input networks, i.e., miRNA similarity network and miRNA-mRNA interaction network.

**FIGURE 3:**
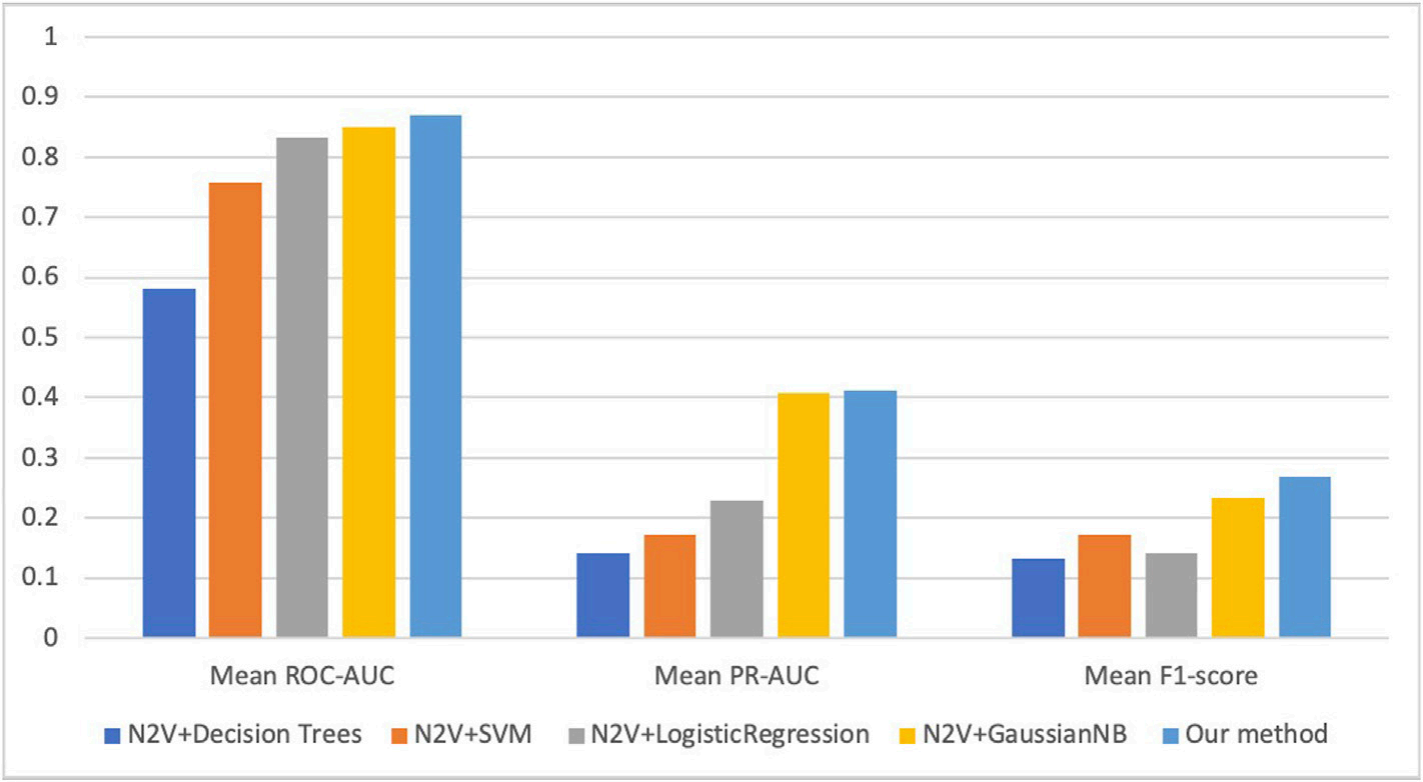
The average ROC-AUC, PR-AUC, and F1-score of five methods on miRNA-MS association prediction task. N2V represents Node2Vec which extracts miRNA features based on two miRNA networks.

Figure 3 shows the results of average ROC-AUC, PR-AUC, and F1-score of five-fold cross-validation in five compared methods. And we can see our CNN-GaussianNB-based method has the best performance in all three metrics, and the GaussianNB-based method has the second-best performance. For the metric of ROC-AUC, our method achieves 0.87, and three other methods, named N2V + SVM, N2V + LogisticRegression, N2V + GaussianNB, achieve >0.75. However, for PR-AUC and F1-score, all these methods achieve less than 0.5 and 0.3 respectively. This is because we use the extremely unbalanced dataset (i.e., using all unlabeled miRNAs as negative samples) for the experiment, and our proposed CNN-GaussianNB-based method still achieves the best performance in such an extremely unbalanced case.

### Effects of miRNA Similarity Network and miRNA-mRNA Interaction Network

In order to verify the necessity of two networks we used to extract features, i.e., the miRNA similarity network and miRNA-mRNA interaction network, we perform prediction tasks separately using different network features. The results are shown in Table 2. It can be seen that when we only use features extracted from the miRNA similarity network, the average ROC-AUC is 0.78. When only the miRNA-mRNA interaction network is used, the average ROC-AUC is 0.82. In our method, we combine node features from two networks, and the average ROC-AUC is 0.87. And the similar situation can be seen in the metrics of PR-AUC and F1-score. We can conclude that combining both network features is more effective for this prediction task than using only features from separate networks.

**TABLE 2:** The average ROC-AUC, PR-AUC, and F1-score of the proposed model using different network features.

Network	Mean ROC-AUC	Mean PR-AUC	Mean F1-score
miRNA similarity network only	0.7824	0.4017	0.2344
miRNA-mRNA interaction network only	0.8222	0.2245	0.2357
Both miRNA networks	0.8696	0.4110	0.2693

### Effect of Feature Extraction Methods

We also evaluate the effect of graph representation methods used for feature extraction. The previous study has shown superior performance in learning latent representations of vertices in a network of DeepWalk (Perozzi et al., 2014) than traditional methods like SpectralClustering (Tang and Liu, 2011), Modularity (Tang and Liu, 2009a), wvRN (Macskassy and Provost, 2003), EdgeCluster (Tang and Liu, 2009b). DeepWalk has a similar strategy as Node2vec. It first generates random paths with fixed length and then uses Skipgram to maximize the co-occurrence probability among nodes that appear within a window in random paths. The main difference between the two methods is that Node2Vec uses a biased random walk strategy to control the walking direction (See Methods). And previous studies have demonstrated that Node2Vec has achieved better performance in many bioinformatics applications (Grover and Leskovec, 2016).

We evaluate the performance of our model in predicting the MS-related miRNA using features extracted by Node2Vec and DeepWalk, respectively. In detail, for Node2Vec, we use same in-out-parameter (q = 0.5) return parameter (p = 10) as in above experiments, and we use other parameters in default in both methods. The results of mean five-fold cross-validation are shown in Table 3. Similarly, in this experiment, we use all unlabeled miRNA-MS associations as the negative samples. We can see that using Node2Vec for feature extraction has better performance than using DeepWalk in all three metrics.

**TABLE 3:** The average ROC-AUC, PR-AUC, and F1-score of the proposed model using different feature extraction methods.

Feature extraction method	Mean ROC-AUC	Mean PR-AUC	Mean F1-score
Node2Vec (Grover and Leskovec, 2016)	0.8696	0.4110	0.2693
DeepWalk (Perozzi et al., 2014)	0.8661	0.3985	0.2408

### Evaluation of Each Network Layer in CNN-Based Framework

In order to verify the necessity of each network layer of the model, we conducted ablation experiments on the model. It can be seen from Table 4 that no matter reducing the convolutional layer, pooling layer or nonlinear transformation layer, the performance of the model will be detrimental to a certain extent. For the ROC-AUC index, the performance drops by 4, 1, and 1% while removing the layer of Relu transformation, the Pooling, and the convolution layer, respectively. For the PR-AUC index, the performance drops by 5, 1, and 3%, coordinately. And for F1-score, the performance drops by 3, 2, and 1%, coordinately. Thus, each network layer in the computational framework contributes significantly in the task of predicting MS-related miRNAs.

**TABLE 4:** The average ROC-AUC, PR-AUC, and F1-score of the proposed model with different layer ablation.

Methods	Mean ROC-AUC	Mean PR-AUC	Mean F1-score
Relu FC-ablation	0.8237	0.3594	0.2404
Pool-ablation	0.8554	0.3992	0.2459
Convolution-ablation	0.8623	0.3751	0.2594
Full pipeline	0.8696	0.4110	0.2693

### Functional Analysis of Top Predicted MS-Related miRNAs

We train the model using all annotated MS-related miRNAs and use the model to predict the probabilities of all rest miRNAs to be MS miRNAs. The top 10 predicted miRNAs are hsa-miR-605-5p, hsa-miR-15b-5p, hsa-miR-16-5p, hsa-miR-17-5p, hsa-miR-181a-5p, hsa-miR-181b-5p, hsa-miR-181c-5p, hsa-miR-18a-3p, hsa-miR-195-5p, hsa-miR-196a-5p. The top 50 miRNAs with the highest prediction probabilities are in Supplementary Table S1. Specifically, miR-15b-5p was identified as a differentially expressed exosomal miRNAs in relapsing-remitting MS patients (Ebrahimkhani et al., 2017). It has been reported that miR-16-5p decreased in PBMCs from MS patients after IFN-β therapy (Hecker et al., 2013). And researchers also found altered expression of miR-17-5p in CD4^+^ lymphocytes of relapsing-remitting MS patients. The miR-181a-5p has been discovered as a prognostic biomarker for amyotrophic lateral sclerosis (Magen et al., 2021). In a recent study (Piotrzkowska et al., 2021), Piotrzkowska et al. observed miR-181b-5p had a 2-fold increase in level in MS patients compared to the control group (*p* < 0.005). The target gene SMAD7 (a negative regulator of TGF-β signaling) of miR-181c-5p has been shown engaged in Th17 cell differentiation, being a major driver of CNS autoimmunity in MS (Zhang et al., 2018).

**FIGURE 4:**
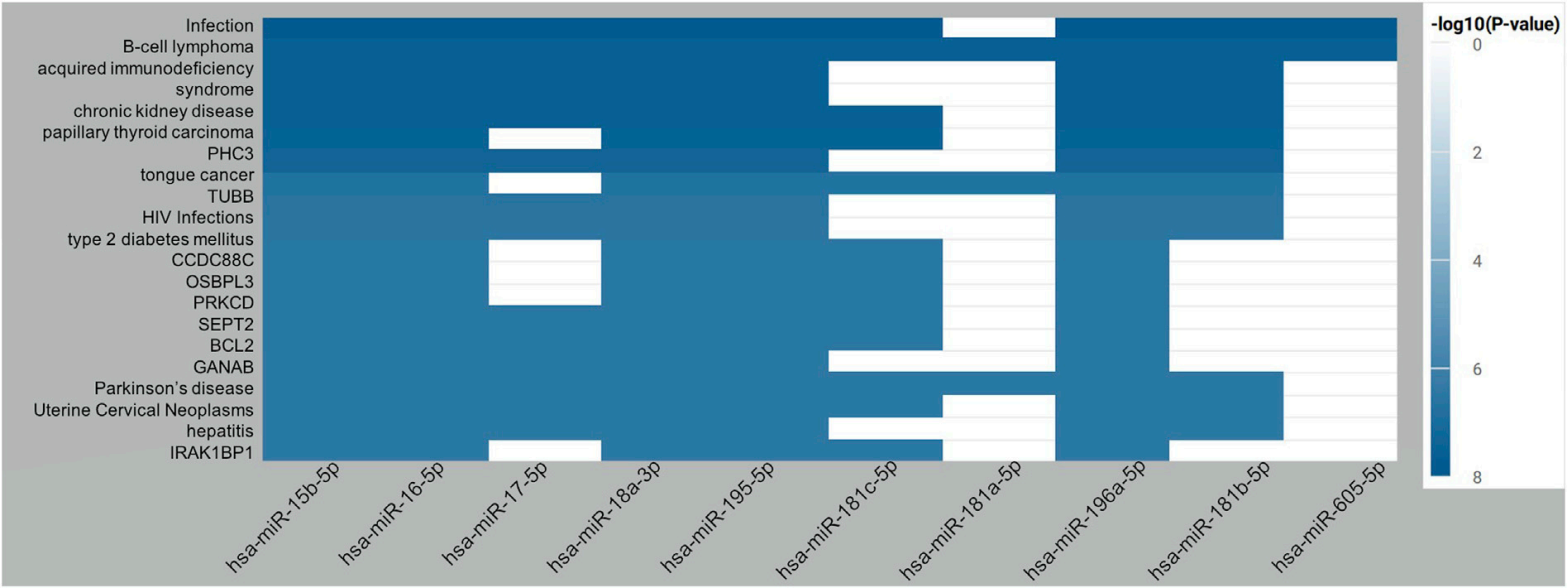
Top twenty enriched terms for the top 10 predicted MS-related miRNAs.

We use miRNA enrichment analysis and annotation tool (miEAA) for functional analysis of the top predicted miRNAs. Over-representation analysis (ORA) is chosen as the enrichment method. Twenty categories are selected for enrichment analysis, such as target genes, diseases, KEGG pathways, Gene Ontology (GO) (see Supplementary Table S2), and FDR (Benjamini-Hochberg) adjustment is used for multiple test correction. Using FDR < 0.05 as the threshold, the full results of enrichment analysis for top predicted MS-related miRNAs are shown in Supplementary Table S2. For illustration, Figure 4 demonstrates the top twenty enriched terms for the top 10 predicted MS-related miRNAs. The best-enriched term is infection with an adjusted *p*-value of 1.43e-8. It has been shown that patients with Multiple Sclerosis have an increased risk of infections compared to the healthy population (Celius, 2017). The second-best enriched term is B-cell lymphoma. A previous study has reported that B-cell lymphoma has some similar symptoms with MS, and early treatment with corticosteroids can improve patient symptoms in both conditions (Lyons et al., 2012). We can also find more genetic or clinical links between Multiple Sclerosis and acquired immunodeficiency syndrome (Morriss et al., 1992), chronic kidney disease (Ruiz-Argüelles et al., 2019), papillary thyroid carcinoma (D’Amico et al., 2019), HIV Infections (Gold et al., 2015), type 2 diabetes mellitus (Hussein and Reddy, 2006), Parkinson’s disease (Delalić et al., 2020), Uterine Cervical Neoplasm (Doosti et al., 2018), and Hepatitis (Cação et al., 2018). Combining our findings, it can be indicated that miRNAs may play important role in various phenotypes in MS patients, and those predicted miRNAs may be potential therapeutic targets for those related diseases and symptoms after wet-lab evaluation.

## Conclusion

Multiple sclerosis is a central nervous system disease that affects a lot of young adults worldwide. For better diagnosis and treatment, it is important to identify the biomarkers of Multiple Sclerosis. Mi-RNA is a type of important biomarker for diseases. However, few computational methods are designed for predicting the Multiple Sclerosis-related miRNAs. In this work, we proposed a computation framework to fill this gap. The proposed framework mainly contains two components, including miRNA features extraction from networks using graph embedding technique and Multiple Sclerosis-related miRNA prediction based on convolutional neural network. Firstly, based on a protein-protein interaction network and a miRNA-gene regulation network, a network representation learning-based method is proposed for feature representation of miRNA. Secondly, taking the low-dimensional features as input, a convolution neural network-based model is proposed for predicting Multiple Sclerosis-associated miRNAs. To demonstrate the advantages of the proposed model, we compare it with several existing methods. The evaluation test shows that the proposed model performs better than existing methods and can predict Multiple Sclerosis-related miRNAs precisely.

## Data Availability

The original contributions presented in the study are included in the article/Supplementary Material, further inquiries can be directed to the corresponding author.
